# Chemical and nutritional composition of some selected lesser known legumes indigenous to Nigeria

**DOI:** 10.1016/j.heliyon.2020.e05497

**Published:** 2020-11-20

**Authors:** Samaila James, Titus Ugochukwu Nwabueze, Gregory I. Onwuka, Joel Ndife, Mohammed Ata'anda Usman

**Affiliations:** a Department of Food Science and Technology, College of Applied Food Sciences, Michael Okpara University of Agriculture, Umudike, Nigeria; b Department of Food Science and Technology, Modibbo Adama University of Technology, Yola, Nigeria

**Keywords:** Food science, Food analysis, Nutrition, Natural product chemistry, Lesser legumes, Minerals, Fibre, Fatty acids, Amino acids

## Abstract

This study evaluated the nutritional composition of Nigeria's lesser known legumes namely African breadfruit (*Treculia africana*), African yam bean (*Sphenostylis stenocarpa*) seed, bambaranut (*Vigna subterranean* L.), red bean (*Phaseolus vulgaris*), groundnut (*Arachis hypogea* L.), African oil bean (*Pentaclethra mycrophylla* Benth.) seed, cowpea (*Vigna unguiculata* (L.) Walp) and pigeon pea (*Cajanus cajan*). The proximate composition, mineral content, fibre profile, fatty acid profile and amino acid compositions were evaluated using standard methods. The results showed that legume samples vary significantly (p < 0.05) in the chemical parameters evaluated. Groundnut, African oil bean seed and African breadfruit had significantly higher protein, carbohydrate, fat and ash contents than other legumes. Equally, groundnut, African oil bean and African breadfruit showed superiority in mineral and fibre abundance, while bambaranut had the lowest mineral and fibre contents. Linolenic acid is the most abundant fatty acid in all the legumes with values ranging from 38.78 – 84.57%. The percentage polyunsaturated fatty acid (PUFA) for all the samples ranged from 40.15 – 48.97%. The total essential amino acids ranged from 24.11 – 66.67 mg/100 g. The range is considered adequate for ideal protein food. Therefore, lesser legumes evaluated can serve as alternative protein sources with good minerals, fibre, essential fatty and amino acids contents.

## 1 Introduction

Legumes belong to the family Leguminosae. In the tropics, legumes are the second most important food crops after cereals and are excellent sources of cheap plant proteins and minerals when compared with animal products (Annor et al., 2014). Indigenous legumes therefore are an important source of affordable alternative protein to poor resource people in many developing countries most especially in Africa and Asia where the grains constitute part of the daily staple food.

Legumes have a special place in the diet of humans, because they contain nearly 2–3 times more proteins than cereals depending on the type (Reyes-Moreno et al., 1993; Annor et al., 2014). Legumes are also excellent sources of complex carbohydrates which have been reported as beneficial for the prevention and management of cardiovascular diseases and diabetes. They also serve as a large reservoir of bioactives, most especially the phenolics (Hu, 2003; Enujiugha, 2010). These bioactives have been positively implicated in the treatment and management of degenerative diseases (Silva et al., 2007; Singh et al., 2017). Furthermore, they are also a good source of vitamins (thiamine, riboflavin, niacin, vitamin pyridoxine and folic acid), minerals (calcium, iron, copper, zinc, phosphorus, potassium and magnesium) and are excellent sources of PUFA (linoleic and linolenic acids) (Ade-Omowaye et al., 2015; Molendi-Coste et al., 2011; Vadivel and Janardhanan, 2005).

To be able to feed the rapidly increasing population in Nigeria, there is need to nutritionally characterize lesser known legumes. Nah and Chau (2010) asserted that there are thousand lesser known plant materials that might substantially add to the array of available nutrients most especially the protein need. These lesser known legumes are well adapted to extreme environmental conditions and highly resistant to drought, diseases and pest infestation. Due to their availability and affordability, hence, the need for such plant materials to be nutritionally characterized for the benefit of human kind most especially in the third world countries where adequate protein intake is a major problem.

In that regard, research studies have been ongoing in presenting lesser known legumes and their suitability in different food applications. Fasoyiro et al. (2006) assessed the proximate, mineral and antinutrient composition of four lesser grains found in Nigeria. The result revealed 22–37% protein, implying their potential in fighting protein deficiencies. The antioxidant properties of some commonly consumed and underutilised legumes in Nigeria were reported (Oboh, 2006). The result revealed high antioxidant and reducing power comparable to known legumes such as soybean. Ade-Omowaye et al. (2015) studied the nutritional potential of nine underexploited legumes in southwest Nigeria. The finding revealed high protein in *Mallotus subulatus* (red variety). While, James et al. (2016) assessed the potentials of protein concentrate from seven legumes indigenous to northern Nigeria for different food applications. The result of the finding showed that, the concentrate has the functionality to be incorporated into different food systems. There is need to investigate other lesser known legumes with dearth of scientific data, hence, the thrust of this study. Therefore, this study evaluated the proximate, minerals, fibre, fatty acids and amino acids compositions of eight lesser known legumes in Nigeria. There proper knowledge would establish their potentials as alternative food sources in counteracting malnutrition.

## 2 Material and methods

### 2.1 Source and preparation of raw materials

African breadfruit (*Treculia africana*) seeds, African yam bean (*Sphenostylis stenocarpa*) seed, bambaranut (*Vigna subterranean* L.), red bean (*Phaseolus vulgaris*), groundnut (*Arachis hypogea* L.), African oil bean (*Pentaclethra mycrophylla* Benth.) seed, cowpea (*Vigna unguiculata* L.) and pigeon pea (*Cajanus cajan*) were procured in the month of January, 2018 from Umuahia Local Market, Abia State, Southeastern Nigeria. The seeds were botanically identified by the Department of Crop Production, Federal University of Technology, Minna, Nigeria. Extraneous matters such as insect infected seed, sand and chaff were manually removed from the samples. Whole seeds of African yam bean, bambaranut, red bean, groundnut, cowpea and pigeon pea were milled in a disc attrition mill (Model No. ED-5, China) and sieved to a particle size of 1 mm. For African oil bean, the seed coats were removed manually prior to milling. While for African breadfruit seeds, the method of James and Nwabueze (2013) was adopted for the removal of seed coat. The seeds were washed in a cold potable water and drained through a local perforated basket. The drained seeds were partially cooked in boiling water for 15 min to facilitate the separation of the seed coats from the endosperm. Partially cooked seeds were drained and allowed to stand for 20 min to further soften the seed coat and to effect cooling. Softened seeds were then decoated in an adjustable disc attrition mill and the endosperm was manually separated from the coat on a tray. The dehulled seeds were oven dried at 60 °C for 17 h and milled to a particle size of 1 mm. The resultant eight flour samples were packaged in a high density polyethylene bag and stored under refrigeration temperature (4 ± 2 °C) until needed for further laboratory analysis.

## 3 Methods

### 3.1 Proximate analyses

#### 3.1.1 Moisture content

The percentage moisture content was determined according to the method described by AOAC (2000). Two gram of the sample was weighed into a petri dish of known weight and the moisture substantially evaporated over water bath. The sample was immediately transferred into an oven and dried at 105 ± 2 °C for 3–5 h. The sample was then removed from the oven and placed in a desiccator to cool for 15 min before weighing. The process was repeated until constant a weight was recorded. The loss in weight from the original weight was reported as the moisture content and calculated using Eq. (1).

(1) $$\text{\% moisture }=\frac{\text{Weight loss}}{\text{Weight of sample taken}}\times 100$$

#### 3.1.2 Extraction of crude fat

Fat content was determined using Soxhlet solvent extraction method. Two gram of the sample labelled A was weighed into the extraction thimble and the thimble was blocked with cotton wool. It was then placed back in the Soxhlet apparatus fitted with a weighed flat bottom flask (B) which was filled to about three quarter of its volume with petroleum ether with boiling point of 40 to 60 °C. The extraction was carried out for a period of 4–8 h for complete extraction. Petroleum ether was removed by evaporation on water bath and the remaining portion in the flask was removed along with water during drying in an oven at 80 °C for 30 min. Defatted sample was then cooled in a desiccator and weighed (C). The percentage fat was calculated using Eq. (2):

(2) $$\text{\% fat }=\frac{\text{Weight of extracted fat }\left(\text{C}-\text{B}\right)}{\text{Weight of sample }\left(\text{A}\right)}\times 100$$

Where; A = Weight of sample; B = Weight of empty flask and C = Weight of flask + oil.

#### 3.1.3 Determination of crude protein

Two gram of the dried sample was transferred into a Kjeldahl flask and 4 g mixture of Na_2_SO_4_/CuS0_4_ and 30 ml concentrated sulphuric acid were added and swirled gently on a heater. The mixture was heated gently at first until frothing stops, then more strongly until a near clear solution resulted. The digested solution was allowed to cool and transferred into a 250 ml volumetric flask. The distillation apparatus was then steamed for 10 min. While this was going on, the volume of the digested solution was made up to the mark and vigorously shaken. Twenty five (25) ml of the digested sample was transferred into Kjeldahl flask and mixed with 25 ml of 40% sodium hydroxide. The mixture of the solution was mounted unto the distillation unit and heated with constant flow of water and the liberated ammonia was collected with 10 ml boric acid-indicator mixture in a conical flask placed at the condenser section of the Markham distillation unit. When the boric acid-indicate mixture turns green, the distillation was allowed to go on for another 5 min. At the end of the time, the conical flask was removed and titrated with 0.01 N hydrochloric acid until the original colour of the boric acid-indicator mixture was restored and percentage nitrogen was quantified using Eq. (3).

(3) $$\text{\% N }=\frac{0.00014\times \text{ titre value }\times 50}{\text{Weight of sample}}\times 100$$

% protein =% N×6.25

#### 3.1.4 Determination of ash content

The weight of the crucible dish was determined. Two gram of the sample was added to the crucible. The dish and its content were placed on the furnace rake and the furnace temperature was set to 500 °C for 16 h until the sample completely turned into ashes. The crucible dish was removed and kept in desiccator to cool and percentage ash was calculated using Eq. (4):

(4) $$\text{\% ash }\frac{\text{Weight of extracted ash}}{\text{Weight of sample}}\times 100$$

#### 3.1.5 Crude fibre determination

Five gram of the sample was weighed into a 500 ml beaker and the content was boiled in 200 ml hydrochloric acid (1%) for 30 min. The suspension was filtered and the residue was washed vigorously with boiling water until it was no longer acidic. The sample residue was then boiled again in a 200 ml sodium hydroxide solution for 30 min, filtered through filter paper (Whatman no.1) and the residue obtained was transferred into a crucible in an air oven 80 °C for 30 min. The dried residue was then cooled in a desiccator and weighed. The weighed sample residue was ashed in a muffle furnace at 550 °C for 30 min. The sample was removed from the furnace when its temperature was 200 °C. It was then cooled in a desiccator and weighed. The loss in weight of the incinerated residue before and after incineration was taken as the crude fibres content and calculated using Eq. (5).

(5) $$\text{\% crude fibre }=\frac{\text{Total weight of fibre}}{\text{Weight of sample}}\times 100$$

#### 3.1.6 Determination of carbohydrate content

Carbohydrate was determined by difference using Eq. (6):

(6) % CHO =100−(% moisture+% protein+% fat+% ash)

#### 3.1.7 Minerals content determination

The mineral contents of legume samples were determined as described by AOAC (2000). The samples were digested by concentrated nitric acid and sulfuric acid (3:1, v/v). Digestion tubes (500 ml) were labelled per sample as well as for the reagent blank (control). One hundred (100 g) gramme of each sample was weighed and placed in a digestion tube. The samples were prepared in triplicates. Five ml concentrated nitric acid was pipetted into each tube. The tubes containing the samples and the reagent blank were set in a digestion block (HYP-308, Shandong, China). The digestion block was turned on and set at 175 °C to start the pre-digestion. The samples were swirled gently twice during the nitric acid pre-digestion, using tongs and protective gloves. The tubes were removed from the digestion block when brown gas started to elute or when solution begins to steam and placed in the cooling rack. Pre-digested samples were allowed to cool for at least 30 min and 4 ml of 30% hydrogen peroxide to each tube was added and gently swirled. The tubes were placed back in the digestion block and the digestion block was turned on and set at 175 °C. Thereafter, the tubes were closely watched for the start of reaction, indicated by the appearance of rolling bubbles. As soon as the reaction started, the tubes were removed from the block and the reaction continued in the cooling rack. The same procedure was repeated for all the samples and the reagent blank.

The tubes were placed back in the digestion block (second phase of digestion) and left until ca. 1–1.5 ml remains, and then removed from the digestion block. The tubes were checked every 10–15 min during this digestion to avoid drying off. Upon attainment of the required stated mile (1–1.5), the tubes were removed cooled and carefully ca. 2 ml concentrated nitric acid was added and continued heating sustained. The digestion block was turned off when all the tubes have been digested and then removed. The samples were filtered using Whatman hardened ashless #540 filter paper into container appropriate for analysis by AAS. For example for sodium (Na) quantification using AAS, ashed sample was diluted with distilled water to 25 ml, then 0.2 ml diluted to 100 ml. The minerals (Fe, K, Na, Zn, Se, Mn, Cu, Ca and Mg) were estimated using an atomic absorption spectrophotometer (210, Buck Scientific USA). Phosphorus (P) was measured by converting phosphates into phosphorus molybdenum blue pigment and measured at 700 nm.

#### 3.1.8 Determination of dietary fibre

The method of AOAC (2000) was adopted for fibre determination. One gram of the sample was weighed and dissolved in ethanol. The prepared sample (microL) was then injected into a High Performance Liquid Chromatography (HPLC) (Buck Scientific BLC10/11, USA) system with a fluorescence detector (with lambda (λ) excitation at 295 nm and lambda (λ) emission at 325 nm) and an analytical column (C18 packed with silica, 25 cm × 4.6 mm, 3 μm particle size, SWASTIK Industries, Vadodara, India) with a stationary nonpolar phase. The mobile phase used was methanol-acetonitrile (v/v) at a flow rate of 1.0 mL/min. Standard samples were also prepared using similar method. Concentration of dietary fibre fractions in samples was calibrated using standards. From the results obtained, the percentages of each sugar fraction in the sample was calculated using Eq. (7):

(7) $$\text{DF }=\frac{\text{A Sample }\times \text{STD }\left(\text{ppm}\right)\times \text{V Met}.\left(\text{ml}\right)}{\left(\text{A STD }\times \text{Wt}.\text{ Sample }\left(\text{g}\right)\right)}\times 100$$

Where: DF = Concentration of dietary fibre in ppm; STD = Concentration of standard; A Sample = Area of sample; A STD = Area of standard; V Met. = Volume of methanol and Wt. Sample = Weight of sample.

#### 3.1.9 Lipid extraction

Total lipid was extracted from the samples in a Soxhlet extractor using n-hexane solvent. Fatty acids were transformed into methyl ester. The lipid sample extracted was placed in a screw-capped glass tube (16.5 × 105 mm) and hydrolyzed with 1 ml of 1 M KOH in 70% ethanol at 90 °C for 1 h. The reaction mixture was acidified with 0.2 ml of 6 M HCl and then 1 ml of water was added. FFAs released were extracted with 1 ml of hexane. After evaporation of the hexane in vacuum, the FFAs were methylated with 1 ml of 10% BF_3_ in methanol at 37 °C for 20 min (AOAC, 2000). The fatty acid methyl esters (FAMEs) were extracted with petroleum ether and were analyzed by high performance liquid chromatograph (HPLC) (Buck Scientific BLC 10/11, USA) equipped with a flame ionization detector and an integrator. The mobile phase was acetonitrile/2-propanol (59:41) and the column (C-18, with 5 μm film thickness, 150 × 4.6 mm and 30 cm length, SWASTIK Industries, Vadodara, India). The flow rate was at 1 mL/min and the oven temperature was maintained at 45 °C for 15 min.

#### 3.1.10 Amino acids determination

The method described by AOAC (2000) was adopted. Amino acid analysis was performed on reverse phase-high pressure liquid chromatography (HPLC) (Buck Scientific BLC 10/11, USA) (15–30 cm) packed with very uniform micro-particulate silica (150 nm, flowrate 1 mL/min, SWASTIK Industries, Vadodara, India). The post column samples were derivatized and data were integrated using peak sample chromatography data system (Buck Sci. Chromatopac Data Processor). Tryptophan was determined spectrophotometrically after alkaline hydrolysis of the samples.

### 3.2 Statistical analysis

The data obtained were in triplicates and the results were subjected to one-way analysis of variance and expressed as mean with standard deviation. The differences between means were separated by Duncan's Multiple Range Test using IBM SPSS Statistics Programme, Version 19.0 (Illinois, USA). Significant differences were expressed at 5% level.

## 4 Result and discussion

### 4.1 Proximate composition of legume samples

Table 1 shows the proximate composition of African breadfruit, bambaranut, red bean, pigeon pea, cowpea, African yam bean, African oil bean, and groundnut. The result shows that legumes samples were all significantly (p < 0.05) different in the proximate parameters measured. The protein, fat, ash, moisture, fibre and carbohydrate contents ranged from 13.25 – 29.34%, 5.87–34.63%, 4.63–9.82%, 9.00–12.75%, 2.23–6.11% and 7.34–64.74% respectively. Groundnut was found to be significantly (p < 0.05) high in protein (29.34%), fat (34.63%), ash (9.82%), moisture (12.75%) and fibre (6.11%). However, it had significantly low carbohydrate (7.34%) compared with other legumes. African oil bean seed ranked next to groundnut in protein, ash, moisture and fibre. Bambaranut was found to be significantly (p < 0.05) low in protein (13.25%), fat (5.87%) ash (4.63%) and fibre (2.23%), however, it was found to be significantly (p < 0.05) high in carbohydrate (64.74%) in comparison with other legume samples.

**Table 1:** Proximate composition of legume samples on dry weight basis (dwb).

Proximate (%)	ABF	BBN	RBS	PGP	CPB	AYB	AOB	GGN
Protein	17.97^c^ ± 0.01	13.25^h^ ± 0.00	14.36^g^ ± 0.01	16.43^f^ ± 0.01	16.85^e^ ± 0.01	17.03^d^ ± 0.01	22.56^b^ ± 0.01	29.34^a^ ± 0.01
Fat	7.00^c^ ± 0.00	5.87^g^ ± 0.01	6.50^f^ ± 0.00	6.73^e^ ± 0.01	6.75^d^ ± 0.00	6.76^d^ ± 0.01	18.50^b^ ± 0.00	34.63^a^ ± 0.01
Ash	6.80^b^ ± 0.00	4.63^d^ ± 0.00	4.72^d^ ± 0.01	4.71^d^ ± 0.72	5.50^c^ ± 0.00	5.53^c^ ± 0.01	6.61^b^ ± 0.01	9.82^a^ ± 0.01
Moisture	9.00^h^ ± 0.00	9.30^f^ ± 0.00	9.25^g^ ± 0.00	9.75^c^ ± 0.00	9.55^d^ ± 0.00	9.50^e^ ± 0.00	11.51^b^ ± 0.01	12.75^a^ ± 0.00
Fibre	3.65^c^ ± 0.00	2.23^h^ ± 0.01	2.50^g^ ± 0.00	3.13^f^ ± 0.01	3.16^c^ ± 0.01	3.29^d^ ± 0.01	5.70^b^ ± 0.00	6.11^a^ ± 0.01
Carbohy	55.89^f^ ± 0.01	64.74^a^ ± 0.02	62.68^b^ ± 0.01	59.76^c^ ± 0.05	58.20^d^ ± 0.01	57.90^e^ ± 0.04	35.12^g^ ± 0.04	7.34^h^ ± 0.05

Values are means and standard deviations of three determinations. Values not followed by the same superscript in the same row are significantly different (p < 0.05).

Key: ABF = African breadfruit, BBN = Bambaranut, RBS = Red bean, PGP = Pigeon pea, CPB = Cowpea, AYB = African yam bean seed, AOB = African oil bean, GGN = Groundnut, Carbohy. = Carbohydrate.

The protein content of African breadfruit obtained in this study 17.97% is high compared with 10.06% and 12. 27% as reported by James and Nwabueze (2013), however, low compared with 25.62% (Nwaigwe and Adejumo, 2015). The protein content of bambaranut determined in this study 13.25%, is low compared with 18.80% and 21.80% reported by Yao et al. (2005) and Hillock et al. (2011) respectively, but, agrees with the findings of Falade and Nwajei (2015). Also, the fibre (2.23%) and fat (5.87%) contents of bambaranut agree with the finding of James et al. (2018). The protein and carbohydrate contents of pigeon pea 16.43% and 59.76% respectively, are within the range reported by Saxena et al. (2010). The protein (16.85%), fibre (3.16%) and carbohydrate (58.20%) contents of cowpea obtained in this study are in agreement with the finding of Adaji et al. (2007). African yam bean protein, fibre and carbohydrate contents 17.03%, 3.29% and 57.90% respectively, are in contrast with the finding of Klu et al. (2001) who reported 19.10% protein, 5.70% fibre and 74.10% carbohydrate. However, the results agree with the findings of Amoetey et al. (2002). Generally, variabilities in the nutrient composition of pulses are attributed to soil types, agronomic practices and genotypes (Uguru and Madukaife, 2001; Nwokeke et al., 2013). The protein (22.56%), fat (18.50%) carbohydrate (35.12%) and fibre (5.70%) contents of African yam bean are in line with the findings of Onwuliri et al. (2004). Groundnut is cherished for its good oil, protein and mineral contents. The protein (29.34%), fat (34.63%) and ash (9.82%) contents agree with the report of USDA (2011).

### 4.2 Mineral composition of legume samples

The mineral composition of legume samples is shown in Table 2. The results show that the eight (8) legumes evaluated vary considerably in all the mineral elements determined. Groundnut had significantly (p < 0.05) high Ca, Fe, Mg, K, P, Cu, Mn, Zn and Se except in sodium (Na) where, African oil bean seed had the highest value. African oil bean seed and African breadfruit, respectively, followed in the abundance of minerals evaluated. The trend of mineral abundance in the legumes can be summarized thus: groundnut > African oil bean > African breadfruit > African yam bean > cowpea > pigeon pea > red bean > bambaranut.

**Table 2:** Mineral composition of legume samples on dry weight basis (dwb).

Minerals (mg/100 g)	ABF	BBN	RBS	PGP	CPB	AYB	AOB	GGN
Calcium	378.34^c^ ± 0.01	185.32^h^ ± 0.01	265.35^g^ ± 0.01	285.43^f^ ± 0.01	294.13^e^ ± 0.01	312.46^d^ ± 0.01	398.60^b^ ± 0.01	415.64^a^ ± 0.02
Magnesium	145.77^c^ ± 0.02	122.55^h^ ± 0.01	134.26^g^ ± 0.01	137.32^f^ ± 0.01	139.65^e^ ± 0.02	141.63^d^ ± 0.01	175.64^b^ ± 0.02	195.92^a^ ± 0.00
Potassium	1243.45^c^ ± 0.02	844.53^h^ ± 0.01	986.35^g^ ± 0.01	1024.54^f^ ± 0.01	1052.39^e^ ± 0.01	1194.06^d^ ± 0.02	1345.8^b^ ± 0.01	1694.36^a^ ± 0.02
Phosphorus	395.66^c^ ± 0.01	131.65^h^ ± 0.01	196.36^g^ ± 0.02	203.15^f^ ± 0.01	242.12^e^ ± 0.01	286.65^d^ ± 0.01	452.27^b^ ± 0.02	526.75^a^ ± 0.00
Sodium	298.66^d^ ± 0.01	177.65^h^ ± 0.01	266.35^g^ ± 0.01	311.46^c^ ± 0.01	298.52^e^ ± 0.01	284.66^f^ ± 0.01	345.76^a^ ± 0.01	336.53^b^ ± 0.01
Manganese	85.33^c^ ± 0.01	35.26^h^ ± 0.01	37.17^g^ ± 0.02	42.19^f^ ± 0.02	44.64^e^ ± 0.01	55.27^d^ ± 0.02	89.27^b^ ± 0.03	95.39^a^ ± 0.03
Iron	12.35^c^ ± 0.01	4.06^h^ ± 0.01	5.65^g^ ± 0.00	6.43^f^ ± 0.01	7.46^e^ ± 0.01	8.46^d^ ± 0.01	15.67^b^ ± 0.02	17.93^a^ ± 0.01
Cupper	4.54^c^ ± 0.02	2.46^h^ ± 0.01	2.86^g^ ± 0.01	3.15^f^ ± 0.00	3.20^e^ ± 0.00	3.35^d^ ± 0.00	7.46^b^ ± 0.01	11.85^a^ ± 0.00
Zinc	25.17^c^ ± 0.01	15.25^h^ ± 0.01	17.12^g^ ± 0.01	18.56^f^ ± 0.01	19.34^e^ ± 0.01	20.16^d^ ± 0.01	35.17^b^ ± 0.03	41.04^a^ ± 0.01
Selenium	0.35^c^ ± 0.01	0.13^g^ ± 0.01	0.16^f^ ± 0.01	0.25^e^ ± 0.01	0.29^d^ ± 0.01	0.33^c^ ± 0.01	0.74^b^ ± 0.01	0.82^a^ ± 0.01

Values are means and standard deviations of three determinations. Values not followed by the same superscript in the same row are significantly different (p < 0.05).

Key: ABF = African breadfruit, BBN = Bambaranut, RBS = Red bean, PGP = Pigeon pea, CPB = Cowpea, AYB = African yam bean seed, AOB = African oil bean and GGN = Groundnut.

Potassium (K) was the most abundant macro element in all the legume samples evaluated. The values ranged from 844.53 to 1694 mg/100 g in groundnut and bambaranut, respectively. Legume samples evaluated have appreciable abundance of other macro elements Ca, Mg, Na and P. Their values ranged from 185.32 – 415.64 mg/100 g, 122.55–195.92 mg/100 g, 177.65–336.53 mg/100 g and 131.65–526.75 mg/100 g, respectively. Mn is the most abundant trace element determined in the legume samples. The values ranged from 35.26 to 95.39 mg/100 g in groundnut and bambaranut, respectively. Other trace elements Fe, Cu and Zn were present in appreciable quantities ranging from 4.06 – 17.93 mg/100 g, 2.46–11 85 mg/100 g and 15.25 and 41.04 mg/100 g, respectively. However, Sn is the least abundant trace element evaluated. The values ranged from 0.31 to 0.82 mg/100 g in bambaranut and groundnut, respectively.

Calcium (Ca) is the most abundant macro element in the human body. In conjunction with phosphorus, they play a role in the process of teeth and bone formation, muscle physiology as well as in the mechanism of blood coagulation (Cormick and Belizán, 2019; Miller et al., 2017). The Ca content ranged from 185.32 – 415.64 mg/100 g, while for phosphorus the content ranged from 131.65 – 526.75 mg/100 g. Groundnut in comparison with other legumes showed superiority in Ca abundance (416.64 mg/100 g). The result of this study agrees with the finding of Settaluri et al. (2012). The Ca content of African oil bean seed (398.60 mg/100 g) and African breadfruit (378.34 mg/100 g), the second and third legumes in Ca abundance are in line with the findings of Akindahunsi (2004). However, bambaranut had significantly (p < 0.05) low Ca (185.32 mg/100 g) and the value agrees with the finding of Yao et al. (2005).

Magnesium (Mg) is an essential macro element needed for normal muscle and nerve functions; regulation of normal blood pressure and blood glucose level and an important element in teeth and bone formation (Gröber et al., 2015; Schwalfenberg and Genuis, 2017). Legumes evaluated have high magnesium content with high abundance in groundnut (195.92 mg/100 g) and low abundance in bambaranut (122.35 mg/100 g). Mg content obtained in this study is in line with the findings of Settaluri et al. (2012) who reported similar range. Also, African oil bean seed, the second legume in Mg abundance and bambaranut the lowest, their respective values agree with the findings of Oyeleke et al. (2014) and Yao et al. (2005).

Potassium (K) is an important element which helps in maintaining body fluid electrolytes balance. In association with sodium ions, potassium plays an important role in the brain and nerve functioning and in muscle development. The range determined 844.50–1694.36 mg/100 g shows that all the legume samples are good food sources. The value in groundnut (1694.36 mg/100 g) is high compared with 658.00 mg/100 g in Indian red groundnut variety (Settaluri et al., 2012). The potassium contents of African oil bean seed (1345.76 mg/100 g) and African breadfruit (1243.45 mg/100 g) in this study are in contrast with the findings of Akindahunsi (2004) and Oyeleke et al. (2014) who reported 235.65–660.50 mg/100 g and 587.00 mg/100 g, respectively. However, the potassium content of African breadfruit strongly agrees with the report of James and Nwabueze (2013). The variations in the mineral contents can be attributed to species differences and source of water during analysis.

Legume samples have considerable amount of iron ranging from 4.06 – 17.93 mg100 g. This shows that all the legumes evaluated are potential sources of iron when consumed in sufficient quantity. Therefore, they can serve as important tools in fighting iron deficiency most especially in the developing countries. The iron content in groundnut (17.93 mg/100 g), African oil bean seed (15.67 mg/100 g) and African breadfruit (12.35 mg/100 g) strongly agree with Onwuliri et al. (2004) and James and Nwabueze (2013) who reported similar findings.

Copper and zinc are important trace elements which play vital roles in the body during metabolisms. They serve as cofactors to a number of key metabolic enzymes (Mustafa and AlSharif, 2018; Uauy et al., 1998). Also, they play important roles in normal growth and development during pregnancy, childhood and adolescence. Their values ranged from 2.46 – 11.85 mg/100 g and 15.25–41.04 mg/100, respectively. The results reveal that lesser legumes under investigation are potential food sources of Cu and Zn. This implies that, the legumes are capable of supplying over 70% and 50% of daily human need of copper and zinc, respectively.

Selenium (Se) is an essential trace element and it serves as a strong antioxidant which helps in the prevention of mutation and possible cancer development (Molendi-Coste et al., 2011; Vadivel and Janardhanan, 2005). Furthermore, it is an essential cofactor to some metabolic enzyme systems. Legume evaluated showed low presence in the range of 0.13–0.82 mg/100 g in bambaranut and groundnut, respectively. This result implies that the selenium content of legumes evaluated is capable of supplying averagely 16% of human daily requirement. Therefore, their consumption in adequate quantity would confer beneficial need to the body.

### 4.3 Fibre profile of legume samples

The interaction of dietary fibre with other bioactive constituents such as polyphenol significantly influence their physiological benefits, besides their fermentation in the large intestine which generates short chain fatty acids that serve as microbial fuel (Singh et al., 2017).

The fibre profile of legume samples is shown in Table 3. The result revealed that all the legume samples studied were significantly (p < 0.05) different in all the fibre profile evaluated. The hemicellulose, raffinose, cellulose, lignin, and starchyose contents ranged from 12.15 – 17.18%, 14.26–18.67%, 38.19–44.09%, 4.03–8.17% and 3.45–6.54% in African breadfruit, bambaranut, red bean, pigeon pea, cowpea, African yam bean, African oil bean and groundnut, respectively. African oil bean had the highest hemicellulose content (17.18%) followed by groundnut (16.43%) and African breadfruit (15.22%) while, bambaranut had the least (12.15%) content. The same trend was observed in raffinose and hemicellulose contents where African oil bean and groundnut had significantly (p < 0.05) high contents. In lignin and starchyose, groundnut had the highest contents 8.17% and 6.54%, respectively. However, red bean (4.16%) and bambaranut (3.45%) showed low lignin and starchyose contents. Dietary fibre is one of the most important bioactive constituents in pulses. Singh et al. (2017) reported that legume seed coat and cotyledons are rich in both soluble and insoluble dietary fibre. The range obtained in this study compares with 14–32% in beans, chickpea, lentils and peas whereas, high compare with 3.00–15.02%, 0.86–4.33%, and 2.14–10.79% in wheat, rice and barley respectively (Singh et al., 2017; Rebello et al., 2014).

**Table 3:** Fibre profile of legume samples on dry weight basis (dwb).

Fibre (%)	ABF	BBN	RBS	PGP	CPB	AYB	AOB	GGN
Hemicell	15.22^c^ ± 0.00	12.15^g^ ± 0.01	13.27^f^ ± 0.01	13.32^e^ ± 0.01	14.26^d^ ± 0.01	14.26^d^ ± 0.01	17.18^a^ ± 0.01	16.34^b^ ± 0.01
Raffinose	14.26^h^ ± 0.01	17.87^c^ ± 0.02	17.64^d^ ± 0.03	17.26^e^ ± 0.01	16.44^f^ ± 0.02	15.65^g^ ± 0.00	18.67^a^ ± 0.01	18.35^b^ ± 0.00
Cellulose	42.14^c^ ± 0.01	38.06^h^ ± 0.00	38.19^g^ ± 0.01	40.21^f^ ± 0.00	41.67^e^ ± 0.01	41.18^d^ ± 0.00	44.09^a^ ± 0.00	43.25^b^ ± 0.01
Lignin	7.08^b^ ± 0.00	4.03^h^ ± 0.01	4.16^g^ ± 0.01	4.19^f^ ± 0.01	5.14^e^ ± 0.01	6.12^d^ ± 0.00	7.04^c^ ± 0.00	8.17^a^ ± 0.01
Starchyose	5.26^c^ ± 0.01	3.45^g^ ± 0.00	3.74^f^ ± 0.02	3.87^e^ ± 0.01	3.95^d^ ± 0.00	3.98^d^ ± 0.01	6.00^b^ ± 0.00	6.54^a^ ± 0.02

Values are means and standard deviations of three determinations. Values not followed by the same superscript in the same row are significantly different (p < 0.05).

Key: ABF = African breadfruit, BBN = Bambaranut, RBS = Red bean, PGP = Pigeon pea, CPB = Cowpea, AYB = African yam bean seed, AOB = African oil bean, GGN = Groundnut, Hemicell. = Hemicellulose.

### 4.4 Fatty acid profile of legume samples

The result of the essential fatty acids composition of the legumes is shown in Table 4. The eight legume samples evaluated showed variations in their fatty acids composition. Groundnut had significantly (p < 0.05) high abundance of all the fatty acids in comparison to other legumes. The value ranged from 28.78 – 84.57 mg/100 g. African oil bean seed ranks second in fatty acid abundance with linoleic having the highest concentration 65.25 mg/100 g while, lauric acid having the lowest concentration (8.45 mg/100 g). African breadfruit ranks third in fatty acid abundance which ranged from 7.23 – 47.87 mg/100 g. African yam bean seed ranks fourth in fatty acids abundance, while bambaranut had the lowest abundance ranging from 3.59 – 38.78 mg/100 g.

**Table 4:** Fatty acid profile of legume samples.

Fatty acid (mg/100 g)	ABF	BBN	RBS	PGP	CPB	AYB	AOB	GGN
Capric (C10:0)	9.21^c^ ± 0.01	3.59^f^ ± 0.01	5.95^e^ ± 0.01	6.54^e^ ± 0.02	6.56^e^ ± 0.00	7.27^d^ ± 0.73	12.46^b^ ± 0.01	39.54^a^ ± 0.02
Lauric (C12:0)	7.23^c^ ± 0.01	5.05^h^ ± 0.01	6.57^g^ ± 0.01	6.63^f^ ± 0.01	6.85^e^ ± 0.00	7.03^d^ ± 0.01	8.45^b^ ± 0.00	28.78^a^ ± 0.01
Myristic (C14:0)	8.47^c^ ± 0.01	3.88^h^ ± 0.01	5.40^g^ ± 0.00	5.54^f^ ± 0.02	6.21^e^ ± 0.01	8.05^d^ ± 0.00	9.56^b^ ± 0.01	30.17^a^ ± 0.02
Palmitic (C16:0)	27.35^c^ ± 0.00	15.52^g^ ± 0.01	16.51^f^ ± 0.01	17.69^e^ ± 0.03	21.34^d^ ± 0.02	21.32^d^ ± 0.00	35.64^b^ ± 0.02	78.76^a^ ± 0.01
Stearic (C18:0)	25.31^c^ ± 0.01	19.54^f^ ± 0.02	17.85^g^ ± 0.00	20.97^e^ ± 0.02	21.64^d^ ± 0.01	21.63^d^ ± 0.00	41.26^b^ ± 0.01	74.15^a^ ± 0.02
Oleic (18:1)	22.21^c^ ± 0.01	19.08^h^ ± 0.01	20.09^g^ ± 0.01	20.49^f^ ± 0.02	21.12^e^ ± 0.01	21.76^d^ ± 0.01	55.46^b^ ± 0.01	76.64^a^ ± 0.02
Linoleic (C18:2)	47.87^c^ ± 0.01	38.78^h^ ± 0.01	39.04^g^ ± 0.01	40.32^f^ ± 0.01	41.61^e^ ± 0.01	42. 54^d^ ± 0.01	65.25^b^ ± 0.01	84.57^a^ ± 0.01
Linolenic (C18:3)	22.66^c^ ± 0.01	18. 42^h^ ± 0.01	19.54^g^ ± 0.02	20.72^f^ ± 0.01	21.21^e^ ± 0.01	21.76^d^ ± 0.01	45.63^b^ ± 0.01	79.86^a^ ± 0.01
Capric (C10:0)	10.01^c^ ± 0.01	6.76^c^ ± 0.01	7.03^c^ ± 0.01	9.62^c^ ± 0.01	10.05^c^ ± 0.01	12.46^c^ ± 0.01	25.65^b^ ± 0.01	55.64^a^ ± 0.01
TEFA	80.54	63.96	65.61	70.66	72.87	76.76	136.53	220.07
%TSFA	43.09	36.42	37.89	38.63	39.98	39.86	35.87	45.87
%TUFA	56.98	63.57	65.61	61.37	60.02	60.14	64.13	54.13
%MUFA	12.32	14.61	14.56	13.80	13.49	13.28	18.53	13.98
%PUFA	44.67	48.97	47.55	47.58	46.54	45.05	45.05	40.15

Values are means and standard deviations of three determinations. Values not followed by the same superscript in the same row are significantly different (p < 0.05).

Key: ABF = African breadfruit, BBN = Bambaranut, RBS = Red bean, PGP = Pigeon pea, CPB = Cowpea, AYB = African yam bean seed, AOB = African oil bean, GGN = Groundnut, TEFA = Total Essential Fatty Acid, %TSFA = Percentage Total Saturated Fatty Acids = %TUFA = Percentage Total Unsaturated Fatty Acid, %MUFA = Percentage Monounsaturated Fatty Acids, %PUFA = Percentage Polyunsaturated Fatty Acids.

Linoleic acid is the most abundant fatty acids in the legumes evaluated and the value ranged from 38.78 - 84.57 g/100 g in groundnut and bambaranut, respectively. This agrees with Ade-Omowaye et al. (2015) who reported similar finding in nine underexploited legume in South West Nigeria. Legume samples evaluated have abundance of palmitic, stearic, oleic and linoleic acids where they ranged from 15.52 – 78.76 mg/100 g, 17.85–74.15 mg/100 g, 19.08–76.64 mg/100 g and 18.42–79.86 mg/100 g, respectively. However, lauric acid is the least abundant fatty acid in the legumes evaluated.

The total fatty acids (TFA) of the legume samples evaluated ranged from 130.62 – 548.11 mg/100 g. The percentage concentrations of total saturated fatty acids (%TSFA) and total unsaturated fatty acids (%TUFA) ranged from 35.87 – 45.87% and 54.13–65.61%, respectively. The result clearly shows that all the legumes evaluated have high percentage total unsaturated fatty acids with groundnut (54.13%) having the lowest value while, AOB (64.13%) having the highest value. African oil bean had the highest percentage total monounsaturated fatty acids (%MUFA) (18.53%). This was followed by bambaranut, red bean, groundnut and pigeon pea which had 14.61%, 14.56%, 13.98% and 13.80%, respectively. However, African breadfruit had the lowest percentage mono unsaturated fatty acids (12.32%). Evaluating the percentage polyunsaturated fatty acids (%PUFA) of the legume samples, it can be deduced that the values are in the same range. However, bambaranut (48.97%) had the highest percentage while groundnut (40.15%) had the lowest percentage. The range obtained in this study is higher than 37.2% in pumpkin (Montesano et al., 2018); however, in agreements with the reports of Ryan et al. (2007) in selected seeds, grains and legumes; and Ade-Omowaye et al. (2015) in nine underexploited legumes of Southwest Nigeria. Polyunsaturated fatty acids have been positively implicated in the treatment and management of degenerative diseases such as cardio vascular disease, neuro degenerative disease, metabolic syndrome, cancer, diabetes arthritis and mental health problems (Molendi-Coste et al., 2011; Ade-Omowaye et al. (2015)). Therefore, good percentages of PUFA in the legume samples evaluated imply positive health benefit when consumed in substantial amount.

### 4.5 Amino acid profile of legume samples

The result of the amino acid profile (Table 5) shows that legume samples investigated vary significantly (p < 0.05) in all the amino acid composition. Groundnut shows superiority in amino acid abundance, while African oil bean seed ranks second. African breadfruit, African yam bean seed, cowpea and pigeon pea compare favourably in amino acid abundance. However, bambaranut and red bean rank low in amino acid abundance.

**Table 5:** Amino acid profile of legume samples.

Amino acid (mg/100g)	ABF	BBN	RBS	PGP	CPB	AYB	AOB	GGN
∗Lysine	5.17^c^ ± 0.01	1.54^h^ ± 0.01	2.62^g^ ± 0.01	3.16^f^ ± 0.01	4.17^e^ ± 0.01	4.83^d^ ± 0.01	5.83^b^ ± 0.01	6.00^a^ ± 0.00
∗Methionine	2.12^c^ ± 0.01	0.86^h^ ± 0.01	1.13^g^ ± 0.01	1.23^f^ ± 0.01	1.32^e^ ± 0.01	1.65^d^ ± 0.01	2.65^b^ ± 0.01	2.78^a^ ± 0.01
∗Threonine	2.66^e^ ± 0.01	1.75^g^ ± 0.01	2.25^f^ ± 0.00	3.41^c^ ± 0.01	2.66^e^ ± 0.01	3.13^d^ ± 0.01	3.83^b^ ± 0.01	3.95^a^ ± 0.00
∗Isoleucine	4.04^c^ ± 0.01	2.01^f^ ± 0.01	2.85^e^ ± 0.01	2.97^d^ ± 0.01	4.04^c^ ± 0.01	5.13^a^ ± 0.01	4.78^b^ ± 0.01	5.16^a^ ± 0.01
∗Leucine	7.93^e^ ± 0.00	6.25^g^ ± 0.01	7.34^f^ ± 0.01	8.13^d^ ± 0.01	7.93^e^ ± 0.00	9.32^c^ ± 0.01	12.41^b^ ± 0.00	13.54^a^ ± 0.02
∗Phenylal.	3.87^d^ ± 0.01	2.75^f^ ± 0.00	3.84^e^ ± 0.01	4.34^c^ ± 0.01	3.87^d^ ± 0.01	5.00^b^ ± 0.00	5.01^b^ ± 0.01	5.15^a^ ± 0.00
∗Valine	7.66^c^ ± 0.01	3.87^h^ ± 0.01	4.01^g^ ± 0.01	4.17^f^ ± 0.01	4.26^e^ ± 0.01	4.52^d^ ± 0.01	7.82^b^ ± 0.01	8.15^a^ ± 0.00
∗Tryptophan	5.26^c^ ± 0.01	2.72^f^ ± 0.01	3.78^e^ ± 0.01	4.11^d^ ± 0.01	5.26^c^ ± 0.01	5.63^b^ ± 0.01	5.63^b^ ± 0.01	6.36^a^ ± 0.01
∗Histidine	3.86^c^ ± 0.01	1.54^f^ ± 0.02	1.87^e^ ± 0.01	2.45^d^ ± 0.00	3.86^c^ ± 0.01	4.04^b^ ± 0.01	4.04^b^ ± 0.01	5.12^a^ ± 0.00
Arginine	6.32^c^ ± 0.00	3.62^g^ ± 0.01	4.24^f^ ± 0.01	4.96^e^ ± 0.02	6.32^c^ ± 0.01	6.24^d^ ± 0.01	7.13^b^ ± 0.01	8.12^a^ ± 0.01
Serine	3.45^e^ ± 0.01	2.80^g^ ± 0.01	3.13^f^ ± 0.01	3.78^d^ ± 0.00	3.45^e^ ± 0.01	4.28^c^ ± 0.01	5.15^b^ ± 0.01	5.77^a^ ± 0.01
Cysteine	2.98^c^ ± 0.02	0.56^g^ ± 0.00	0.70^f^ ± 0.00	0.97^e^ ± 0.02	0.98^e^ ± 0.01	1.77^d^ ± 0.01	3.66^b^ ± 0.01	4.65^a^ ± 0.00
Tyrosine	5.82^c^ ± 0.01	3.23^g^ ± 0.15	4.62^f^ ± 0.01	5.65^d^ ± 0.02	5.82^c^ ± 0.01	5.37^e^ ± 0.01	7.13^b^ ± 0.00	7.15^a^ ± 0.01
Alanine	6.15^d^ ± 0.00	1.77^h^ ± 0.00	2.72^g^ ± 0.00	3.71^f^ ± 0.01	4.15^e^ ± 0.01	6.54^c^ ± 0.00	7.56^b^ ± 0.01	8.25^c^ ± 0.01
Aspartic A.	6.35^g^ ± 0.01	4.57^h^ ± 0.01	6.52^f^ ± 0.03	7.14^e^ ± 0.10	7.35^d^ ± 0.01	8.23^a^ ± 0.01	7.65^c^ ± 0.00	8.01^b^ ± 0.01
Glutamic A	11.11^c^ ± 0.01	4.43^d^ ± 0.00	4.14^h^ ± 0.00	4.67^g^ ± 0.01	4.11^f^ ± 0.01	4.39^e^ ± 0.01	12.46^b^ ± 0.01	13.29^a^ ± 0.01
Glycine	6.05^c^ ± 0.00	3.13^g^ ± 0.02	3.42^f^ ± 0.01	4.42^e^ ± 0.01	6.05^c^ ± 0.01	5.99^d^ ± 0.00	9.26^b^ ± 0.01	11.43^a^ ± 0.01
Proline	3.28^c^ ± 0.03	1.71^g^ ± 0.00	1.83^f^ ± 0.02	2.28^e^ ± 0.01	2.28^e^ ± 0.01	2.35^d^ ± 0.01	5.55^b^ ± 0.01	6.25^a^ ± 0.00
TAA	90.8	47.40	59.18	69.27	75.60	86.06	112.00	122.88
TEAA	42.57	23.29	29.69	33.97	37.37	43.25	52.00	56.21
TNEAA	48.23	24.11	29.49	35.30	38.23	42.81	60.00	66.67
%TNEAA	53.12	50.86	49.83	50.96	50.57	49.74	53.57	54.26
TSulfurAA	5.10	1.42	1.83	2.20	2.30	3.42	6.31	7.43
%CyInTSAA	58.43	39.44	38.25	44.10	42.61	51.75	58.00	62.58
TEAA/TNEAA	0.88	0.97	1.01	0.96	0.98	1.01	0.87	0.84
TArAA	9.69	5.98	8.46	9.99	9.69	10.37	12.14	12.30
TAcidicAA	17.46	9.00	10.66	11.81	11.46	12.62	20.11	21.30
TBasicAA	15.35	6.70	8.73	10.57	14.35	15.11	17.00	19.24
TNeutralAA	57.99	31.70	39.79	46.89	49.79	58.33	74.89	82.34

Values are means and standard deviations of three determinations. Values not followed by the same superscript in the same row are significantly different (p < 0.05).

Key: ∗ = Essential amino acids, ABF = African breadfruit, BBN = Bambaranut, RBS = Red bean, PGP = Pigeon pea, CPB = Cowpea, AYB = African yam bean seed, AOB = African oil bean, GGN = Groundnut, Aspartic A. = Aspartic Acid, Glutamic A = Glutamic Acid, TAA = Total Amino Acids, TEAA = Total Essential Amino Acids, TNEAA = Total Nonessential Amino Acids, %TNEAA = Percentage Total Nonessential Amino Acids, TSulfurAA = Total Sulfur Amino Acids, %CysInTSAA = Percentage Cysteine In Total Sulfur Amino Acids, Ratio of TEAA/TNEAA = Ratio of Total Essential Amino Acids/Total Nonessential Amino Acids, TArAA = Total Aromatic Amino Acids, TAcidicAA = Total Acidic Amino Acids, TBasicAA = Total Basic Amino Acids, TNeutralAA = Total Neutral Amino Acids.

Glutamic acid is the most abundant amino acid in all the samples evaluated with groundnut having significantly (p < 0.05) high value (13.29 mg/100 g), while red bean had the lowest value (4.14 mg/100 g). The abundance of glutamic amino acids in legume samples evaluated agrees with the findings of Ade-Omowaye et al. (2015) and Vadivel and Janardhanan (2005) who reported similar abundance in Indian wild legumes and under exploited legumes of South West Nigeria. Leucine is the second most abundant amino acid evaluated. The values ranged from 6.25 – 13.54 mg/100 g in groundnut and bambaranut, respectively. This result contradicts the findings of Ade-Omowaye et al. (2015) who reported aspartic acid as the second most abundant amino acid in nine (9) under exploited legume of South West Nigeria.

Leucine is the most abundant essential amino acid in all the samples evaluated with values ranging from 13.54 to 6.25 mg/100 g. This result agrees with the findings of Ogunbusola et al. (2010) and Ade-Omowaye et al. (2015) who reported similar findings in *Lagenaria siceraria* seed flour and nine underutilised legumes of South West Nigeria. Legume samples evaluated showed good spread of all the essential amino acids. However, sulphur containing amino acids methionine and cysteine were low in abundance with values ranging from 0.86 – 2.78 mg/100 g and 0.56–4.65 mg/100 g, respectively. Sulfur containing amino acids are the limiting amino acids in legumes. Their ranges obtained in this study are high compared with 0.11 mg/100 g in chick pea field pea, green pea, lentils and common beans (Wang and Daun, 2004; Iqbal et al., 2006). Legumes are often complemented with cereals, which are rich in sulfur containing amino acids in order to meet the body's amino acid dietary requirement (James et al., 2016).

The total amino acid (TAA) contents were between 47.40 – 122.88 mg/100g. The total essential amino acids (TEAA) of the legume samples ranged from 23.29 – 56.21 mg/100 g. The range of total essential amino acids obtained in this study is low compared with 89.00–236.00 mg/100 g in seven important legumes, however, compares favourably with 45.53–48.44 mg/100 g in nine underexploited legume indigenous to South West Nigeria (Ade-Omowaye et al., 2015). The range of the total essential acid obtained in this study is well above 36% adequate ideal protein recommended by FAO (2002). Legume samples evaluated have nearly equal percentage distribution of essential and non-essential amino acids, except in African breadfruit, African oil bean seeds and groundnut who had their percentage total non-essential amino acids slightly above 50%. The total sulfur amino acids of the legumes evaluated ranged from 1.42 – 7.43 mg/100 g. The percentage cysteine ranged from 38.25 – 62.58%. Total aromatic amino acids were in the range of 5.98–12.30 mg/100 g. The samples showed good amount of aromatic amino acids, however, bambaranut had the lowest value (5.98 mg/100 g). This result implies that legumes evaluated are potential sources of aroma compounds. The ranges of total acidic, basic and neutral amino acids were 9.00–21.30mg/100 g, 6.70–19.24 mg/100 g and 31.70–82.34 mg/100 g, respectively. These ranges imply that proteins of the legumes evaluated might be acidic in nature with exception of cowpea and African yam bean seed who might likely have proteins that are basic in nature. The results of this findings agree with Ogunbusola et al. (2010) and Ade-Omowaye et al. (2015) who reported similar observations in some legumes indigenous to southwest Nigeria.

## 5 Conclusion

Lesser known legumes evaluated differ significantly in their chemical and nutritional compositions. Groundnut, African oil bean seed and African breadfruit showed superiority in protein, carbohydrate, fat and ash contents. Also, in minerals and fibre abundance, groundnut, African oil bean and African breadfruit had high contents while, bambaranut showed the values. In fatty acid composition, linolenic acid is the most abundant fatty acid in all the legumes with values ranging from 38.78 – 84.57%; while, the percentage polyunsaturated fatty acid (PUFA) for all the samples ranged from 40.15 – 48.97%. The total essential amino acids ranged from 24.11 – 66.67 mg/100 g. The range is considered adequate for ideal protein food. Glutamic acid is the most abundant amino acid in all the samples evaluated while, leucine is the most abundant essential amino acid in all the samples evaluated with values ranging from 13.54 to 6.25 mg/100 g.

## Declarations

### Author contribution statement

Samaila James: Conceived and designed the experiments; Performed the experiments; Analyzed and interpreted the data; Contributed reagents, materials, analysis tools or data; Wrote the paper.

Titus Ugochukwu Nwabueze, Gregory I. Onwuka: Conceived and designed the experiments; Contributed reagents, materials, analysis tools or data; Wrote the paper.

Joel Ndife: Performed the experiments; Contributed reagents, materials, analysis tools or data; Wrote the paper.

Mohammed Ata’anda Usman: Analyzed and interpreted the data; Contributed reagents, materials, analysis tools or data; Wrote the paper.

### Funding statement

This research did not receive any specific grant from funding agencies in the public, commercial, or not-for-profit sectors.

### Data Availability Statement

Data included in article/supplementary material/referenced in article.

### Declaration of interests statement

The authors declare no conflict of interest.

### Additional information

No additional information is available for this paper.
